
JOURNAL INFORMATION
==============================
NLM Title Abbreviation: ScientificWorldJournal
Journal ID: ScientificWorldJournal
NLM Journal ID: 101131163
Journal ID: TSWJ

The Scientific World Journal

ISSN: 2356-6140
EISSN: 1537-744X
Publisher: Wiley

ARTICLE INFORMATION
==============================
PMCID: PMC6009407
PMID: 29973815
DOI: 10.1100/tsw.2002.41
Article ID: 575360
Article version: 1
Subjects: Short Report

The Mammalian Cell as a Macromolecular Assembly

Deutscher Murray P. *
Hudder Alice
Nathanson Lubov
Stapulionis Romualdas
Department of Biochemistry and Molecular Biology, University of Miami School of Medicine, P.O. Box 016139, Miami FL 33101, USA
*Murray P. Deutscher: mdeutsch@med.miami.edu
Publication date: 2002
Electronic publication date: 2002 Jan 29
Volume: 2
First page: 87
Last page: 88
Copyright: Copyright © 2002 Murray P. Deutscher et al.
Copyright year: 2002
License: This is an open access article distributed under the Creative Commons Attribution License, which permits unrestricted use, distribution, and reproduction in any medium, provided the original work is properly cited.
License URL: https://creativecommons.org/licenses/by/3.0/

Funding: National Institutes of Health GM16317

==============================
Advances in Gene Technology: The Genome and Beyond –
Structural Biology for Medicine (Proceedings of the 2002 Miami
*Nature Biotechnology* Winter Symposium)
*TheScientificWorld* 2002, 2(S2), 87–88
ISSN 1532-2246; DOI 10.1100/tsw.2002.41

### THE MAMMALIAN CELL AS A MACROMOLECULAR ASSEMBLY

Murray P. Deutscher, Alice Hudder, Lubov Nathanson, and Romualdas Stapulionis
Department of Biochemistry and Molecular Biology, University of Miami School of Medicine,
P.O. Box 016139, Miami FL 33101
mdeutsch@med.miami.edu

**INTRODUCTION.** Throughout much of the history of biochemistry a reductionist approach has dominated attempts to understand cell function[1]. While such studies have been essential for identifying cell components and determining their catalytic and structural properties, by necessity, this approach destroys the structural organization that distinguishes a living cell from a test tube containing the same components. In recent years, it has become evident that interactions among macromolecules play an important role in the efficiency of cellular metabolism, and various methodologies have been employed to study such interactions. We have been investigating the mammalian protein synthetic machinery as a model system to understand the importance of structural organization for *in vivo* cell function.

**METHODS.** Chinese hamster ovary cells, permeabilized with the plant glycoside, saponin, were used to measure protein synthesis and to study leakage of macromolecular components. These cells retain much of the structural organization of living cells.

**RESULTS.** Protein synthesis in permeabilized cells proceeds at rates approaching those of intact cells, and is many times higher that that of usual cell-free systems[2]. Efficient protein synthesis in permeabilized cells requires the addition of $K^+$, $Mg^{2+}$, and creatine phosphate, but macromolecular components are retained within the cell and do not have to be supplied from the outside. As observed with intact cells, aminoacyl-tRNA is channeled in this system, being directly transferred from one component of the translation system to the next, but is absent from the cell fluid where it could leak from the permeabilized cell. Disruption of the microfilament, but not the microtubule, network of the cell, dramatically decreases protein synthesis and leads to leakage of aminoacyl-tRNA and a variety of other translation components. Extension of these studies to other cellular macromolecules reveals that they behave similarly to the translation machinery.

**DISCUSSION**. Based on simple experiments monitoring leakage from permeabilized cells, we conclude that the protein synthetic machinery, and other macromolecules as well, are sequestered within cells and do not diffuse in the cell fluid. The cytoskeletal network plays an important role in maintaining the structural organization of the cell. The data suggest that the majority of macromolecules are directly or indirectly bound to cytoskeletal (and membranous) elements within cells, and probably move by motor-driven processes, rather than by diffusion.

**ACKNOWLEDGMENT.** This work was supported by Grant GM16317 from the National Institutes of Health.

### REFERENCES

1. Srere, P. (2000) Trends Biochem. Sci. 25, 150–153.
2. Deutscher, M.P. (2000) in Technological and Medical Implications of Metabolic Control Analysis. Cornish-Bowden, A.J. and Cardenas, M.L., Eds. Kluwer Publishers, Dordrecht, Netherlands. pp. 215–221.
